# Supplementary material for: An Automated Open-Source Workflow for Standards-Compliant Integration of Small Animal Magnetic Resonance Imaging Data
Source: Front Neuroinform. 2020 Feb 11;14:5. doi: 10.3389/fninf.2020.00005 (PMC7026488; doi:10.3389/fninf.2020.00005)
Supplement: Supplementary file 1 [file Presentation_1.pdf]

Supplementary Materials

```
user@host ~ $ SAMRI bru2bids --help
usage: SAMRI bru2bids [-h] [-b BIDS_EXTRA] [--dataset-authors DATASET_AUTHORS] [--dataset-license DATASET_LICENSE] [--dataset-name]
                      [--debug] [-d DIFFUSION_MATCH] [-e EXCLUDE] [-f FUNCTIONAL_MATCH] [-i] [--keep-crashdump] [--keep-work]
                      [-m [MEASUREMENTS [MEASUREMENTS ...]]] [-n N_PROCS] [-o OUT_BASE] [-s STRUCTURAL_MATCH] [-w WORKFLOW_NAME]
                      measurements-base

   Convert and reorganize Bruker "raw" directories (2dseq and ParaVision-formatted metadata files) into a BIDS-organized file hierarchy containing
   NIfTI files and associated metadata. If any exist, this workflow also reposits COSplay event files (already written according to BIDS) in the
   correct place in the output hierarchy.

   Parameters
   ----------

   measurements_base : str
      Path of the top level directory containing all the Bruker ParaVision scan directories to be converted and reformatted.
   bids_extra : list, optional
      List of strings denoting optional BIDS fields to include in the resulting file names. Accepted items are 'acq' and 'run'.
   dataset_authors : list of string, optional
      A list of dataset author names, which will be written into the BIDS metadata file. Generally not needed, unless this is important for you.
   dataset_license : string, optional
      A dataset license name that will be written into the BIDS metadata file. Generally not needed, unless this is important for you.
   dataset_name : string, optional
      A dataset name that will be written into the BIDS metadata file. Generally not needed, as by default we use the dataset path to satisfy this
      BIDS requirement.
   debug : bool, optional
      Whether to enable debug support. This prints the data selection before passing it to the nipype workflow management system, and turns on debug
      support in nipype (leading to more verbose logging).
   diffusion_match : dict, optional
      A dictionary with any combination of "session", "subject", "task", and "acquisition" as keys and corresponding lists of identifiers as values.
      Only diffusion scans matching all identifiers will be included - i.e. this is a whitelist.
   exclude : dict, optional
      A dictionary with any combination of "session", "subject", "task" , and "acquisition" as keys and corresponding identifiers as values.Only scans
      not matching any of the listed criteria will be included in the workfolow - i.e. this is a blacklist (for functional and structural scans).
   functional_match : dict, optional
      A dictionary with any combination of "session", "subject", "task", and "acquisition" as keys and corresponding lists of identifiers as values.
      Only functional scans matching all identifiers will be included - i.e. this is a whitelist.
   inflated_size : bool, optional
      Whether to inflate the voxel size reported by the scanner when converting the data to NIfTI. Setting this to `True` multiplies the voxel edge
      lengths by 10 (i.e. the volume by 1000); this is occasionally done in some small animal pipelines, which use routines designed exclusively for
      human data. Unless you are looking to reproduce such a workflow, this should be set to `True`.
   keep_crashdump : bool, optional
      Whether to keep the crashdump directory (containing all the crash reports for intermediary workflow steps, as managed by nipypye). This is
      useful for debugging and quality control.
   keep_work : bool, optional
      Whether to keep the work directory (containing all the intermediary workflow steps, as managed by nipypye). This is useful for debugging and
      quality control.
   measurements : list, optional
      Whitelist of Bruker ParaVision scan directories to consider.
   n_procs : int, optional
      Maximum number of processes which to simultaneously spawn for the workflow. If not explicitly defined, this is automatically calculated from the
      number of available cores and under the assumption that the workflow will be the main process running for the duration that it is running.
   out_base : str, optional
      Base directory in which to place the BIDS reposited data. If not present the BIDS records will be created in the `measurements_base` directory.
   structural_match : dict, optional
      A dictionary with any combination of "session", "subject", "task", and "acquisition" as keys and corresponding lists of identifiers as values.
      Only structural scans matching all identifiers will be included - i.e. this is a whitelist.
   workflow_name : str, optional
      Top level name for the output directory.

positional arguments:
  measurements-base     -

optional arguments:
  -h, --help            show this help message and exit
  -b BIDS_EXTRA, --bids-extra BIDS_EXTRA
                        ['acq', 'run']
  --dataset-authors DATASET_AUTHORS
                        []
  --dataset-license DATASET_LICENSE
                        ''
  --dataset-name        False
  --debug               False
  -d DIFFUSION_MATCH, --diffusion-match DIFFUSION_MATCH

  -e EXCLUDE, --exclude EXCLUDE

  -f FUNCTIONAL_MATCH, --functional-match FUNCTIONAL_MATCH

  -i, --inflated-size   False
  --keep-crashdump      False
  --keep-work           False
  -m [MEASUREMENTS [MEASUREMENTS ...]], --measurements [MEASUREMENTS [MEASUREMENTS ...]]
                        []
  -n N_PROCS, --n-procs N_PROCS
                        2
  -o OUT_BASE, --out-base OUT_BASE
                        -
  -s STRUCTURAL_MATCH, --structural-match STRUCTURAL_MATCH

  -w WORKFLOW_NAME, --workflow-name WORKFLOW_NAME
                        'bids'
```

Figure S1: Example of the SAMRI `bru2bids` repositing workflow parameter list as reported in the Bash command line in the `0.4.1` SAMRI release. This output is autogenerated for Bash from the Python docstring.

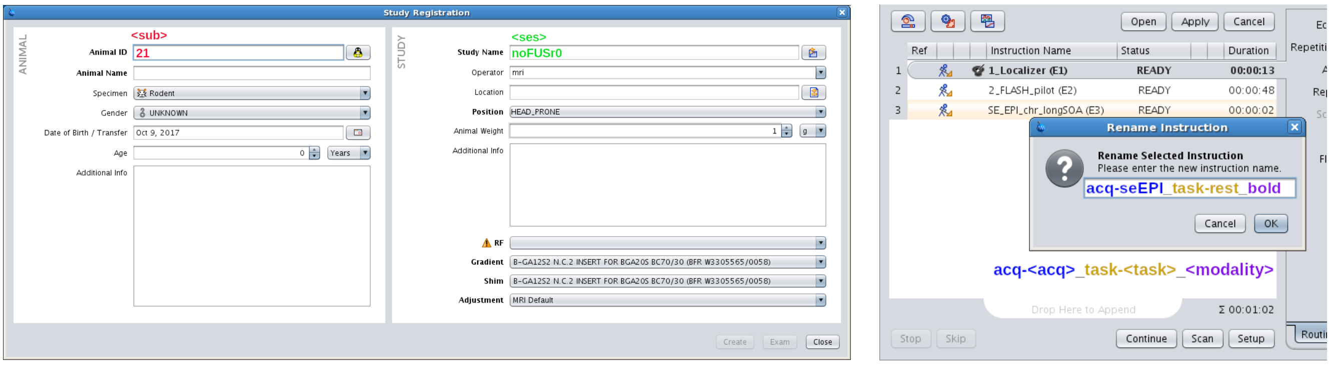

(a) Subject and session are entered into the "Study Registration" window, which is presented to the user before the onset of acquisition.

(b) Other operator input fields are entered into the "Instruction Name" column.

Figure S2: ParaVision graphical user interface examples, showing how metadata automatically interpretable by the repositing workflow can be specified when acquiring new data.
